# Thermal pace-of-life strategies improve phenological predictions in ectotherms

**DOI:** 10.1038/s41598-018-34274-1

**Published:** 2018-10-26

**Authors:** Quentin Struelens, François Rebaudo, Reinaldo Quispe, Olivier Dangles

**Affiliations:** 1 Muséum National d’Histoire Naturelle, Sorbonne Universités, Paris, France; 2 Institut de Recherche pour le Développement, Centre d’Ecologie Fonctionnelle et Evolutive, UMR 5175, CNRS, Université de Montpellier, Université Paul Valéry, Montpellier, EPHE, IRD, Montpellier, France; 3 Institut de Recherche pour le Développement, UMR EGCE-Université Paris Sud-CNRS-IRD-Paris Saclay, Gif-sur-Yvette, France; 4 Fundación PROINPA, La Paz, Bolivia; 5 Department of Ecology and Evolutionary Biology, Cornell University, Ithaca, New York USA

## Abstract

Phenological variability among populations is widespread in nature. A few predictive phenological models integrate intrapopulational variability, but none has ever explored the individual strategies potentially occurring within a population. The “pace-of-life” syndrome accounts for such individual strategies, but has yet to be explored under a phenological context. Here we integrated, for the first time, the slow-fast thermal strategies stemming from the “pace-of-life” into a mechanistic predictive framework. We obtained 4619 phenological observations of an important crop pest in the Bolivian Andes by individually following 840 individuals under five rearing temperatures and across nine life stages. The model calibrated with the observed individual “pace-of-life” strategies showed a higher accuracy in phenological predictions than when accounting for intrapopulational variability alone. We further explored our framework with generated data and suggest that ectotherm species with a high number of life stages and with slow and/or fast individuals should exhibit a greater variance of populational phenology, resulting in a potentially longer time window of interaction with other species. We believe that the “pace-of-life” framework is a promising approach to improve phenological prediction across a wide array of species.

## Introduction

Over the last decades, climate change has induced an advance in phenological events for many plant and animal species^1–3^. Accurately predicting phenological changes is challenging because of the interactions between numerous causal factors driving phenological events^4–6^. Among these factors, phenological responses between individuals can account for a significant amount of variation in phenology within populations, even though this variation has seldom been studied^7^. A non-exhaustive review of existing literature (Supplementary Table S1) reveals that phenological differences between individuals are a widespread phenomenon across taxa, and are in part explained by the mosaic nature of the environment, with each individual experiencing a unique set of environmental conditions^8,9^. Inter-individual differences in phenology also occur under controlled conditions, suggesting that physiological drivers are also involved. In particular, inter-individual differences in development affect phenology because the duration of a life stage at the population level depends on (i) the inter-individual differences in synchrony of the life stage, and (ii) the inter-individual differences in time needed to complete the stage^4,10^. Even though some phenological models integrate intrapopulational variation in development^11,12^, none has ever explored the individual strategies potentially occurring within a population.

The “pace-of-life” (POL) syndrome postulates that a population can be divided into subpopulations with “fast” and “slow” individuals reflecting their life-history strategies and adaptations to different environmental conditions^13^. Each pace displays several correlated traits, such as energy expenditure, growth rate or lifespan^14^. Such individual strategies in performance rate occurs in natural population and has been reported for several species of trees^15^, birds^16,17^, mammals^18–20^ and invertebrates^21,22^. However, POL strategies have seldom been applied to development studies (but see^22^) as this requires the individual following of several populations over their entire life cycle. Life cycles of most organisms include different periods (referred as life stages) throughout their ontogeny, with stage-specific form, lifestyle, reproductive capacity, or physiology^10,23,24^. Thus, differences in development rate not only occur between individuals, but also between life stages within an individual’s life cycle. The POL syndrome could be divided across the several life stages, with faster and slower individuals for a specific life stage. However, it is unclear whether the development strategies are consistent across life stages^22^. Surprisingly, little attention has been paid to the incorporation of POL strategies in phenological predictions, despite a novel focus on POL strategies in other performances^15–21^.

Inter-individual differences in thermal performances have been suggested as a source of the POL syndrome, through their effects on metabolism^25^. Ectothermic organisms are ideal models to study this relationship as environmental temperature constrains several physiological performances including development that acts as a major determinant of the individual’s phenology^26,27^. Moreover, each life stage exhibits different ranges of performance responses to temperature (usually characterized by thermal performance curves) that reflects its adaptation to a specific habitat^28^. Even when experiencing constant temperatures, ectotherms show variability in emergence dates, owing to differences in development rate across individuals and life stages. However, when submitted to fluctuating temperatures, complex interactions can arise between the physiological differences and the temperatures experienced, with potential lags and accelerations in development time between individuals^10^. Lag between individuals implies that different temperatures can be experienced during a specific development event, which has long-lasting consequences on future development and metabolism^29,30^.

We hypothesize that taking into account thermal POL strategies in development rate models would improve phenological predictions in ectotherms. To address this issue our objectives are to: (i) determine the occurrence of thermal POL strategies in development, (ii) develop and assess a new framework accounting for thermal POL strategies in phenological models, and (iii) explore the effect of population slow-fast strategies composition on phenological predictions.

**Figure 1:**
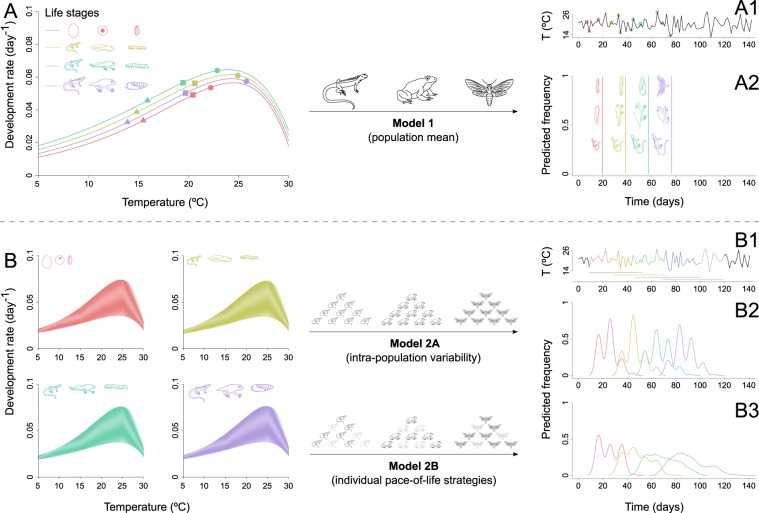
Model construction and theoretical predictions of the phenology of an ectotherm species (e.g., lizard, frog or moth) at different life stages (e.g., egg, pre-larva, larva and pupa) based on simulated thermal performance data and temperature time-series experienced over the life cycle. Model 1 fits a mean thermal performance curve (TPC) between temperature and species’ development rates for the four life stages (**A**), and then applies the TPC over a temperature time series (A1; circles, squares and triangles illustrate the correspondence between the TPC and the temperature time-series) to obtain the phenology of the four life stages (A2). Model 2A constructs a thermal performance probability (TPP) surface for the four life stages (**B**) and then applies the TPP over the same temperature time-series (B1) to obtain the population distribution of the phenology over time (B2), resulting in overlapping life stages over time (B1,B2). Model 2B applies the same TPP than Model 2A (**B**) over the temperature time series (B1) but takes into account slow-fast strategies in development among individuals (see text for details) to obtain population distribution of the phenology over time (B3). Note that species are represented for illustrative purpose and do not depict realistic development rates.

We developed three individual-based models following a growing complexity, and compared them using real development data from *Copitarsia incommoda*. Model 1 employs the commonly-used thermal performance curve that estimates a continuous mean performance rate for the whole population^31^, resulting in identical phenology across individuals (Fig. 1A2). Model 2A overcomes this issue by capturing a range of development rates naturally occurring within a population (Fig. 1B). Finally, Model 2B accounts for individual slow-fast thermal strategies (Fig. 1C; see Methods for details about the models).

## Results

### Models’ predictions comparison: predicting the phenology of the quinoa moth

**Figure 2:**
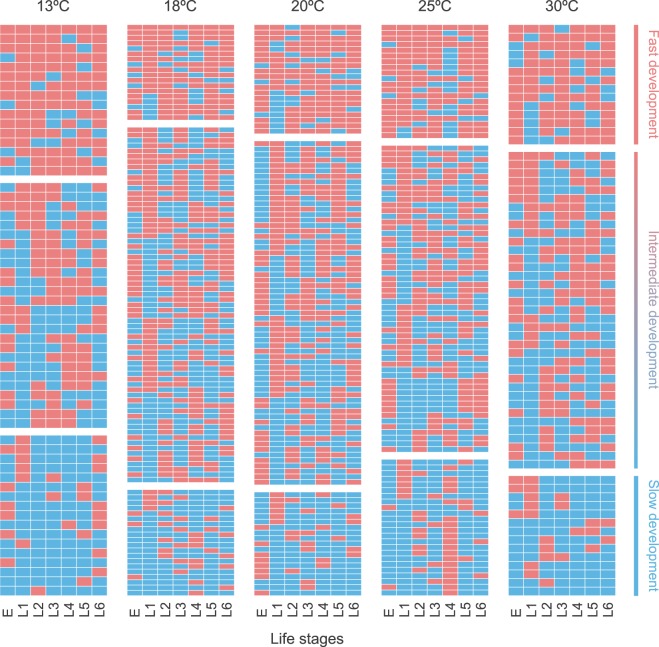
Shifts in pace-of-life development strategies observed within the population of *C. incommoda* across life stages, for five populations reared at 13 °C, 18 °C, 20 °C, 25 °C and 30 °C. Each row represents an individual followed separately across all life stages (in column, E = eggs, and L1 to L6 = larval instars). A red square means that the individual development rate stands in the upper half (fast strategy) for the considered life stage, while the blue square stands in the lower half (slow strategy). We defined the slow individuals (29%, 19%, 18%, 24% and 22% of the populations reared at 13, 18, 21, 25, and 30 °C, respectively) and fast individuals (30%, 17%, 19%, 20% and 22% of the populations reared at 13, 18, 21, 25, and 30 °C, respectively) as individuals that stayed within the same category at least five out seven life stages (separated by white spaces).

**Figure 3:**
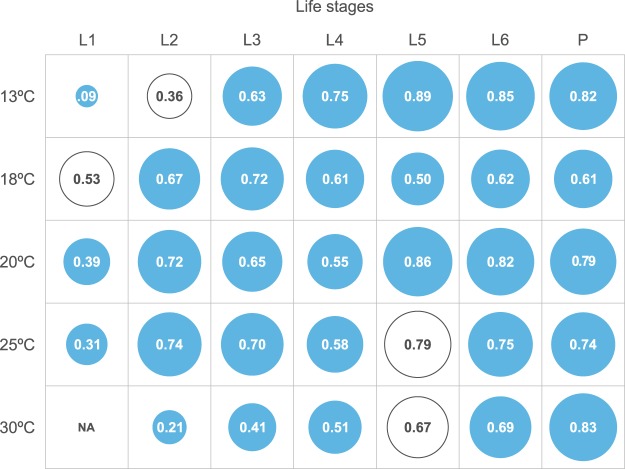
Best overlap scores between predicted and observed phenology distributions of *C. incommoda* life stages at five temperatures. White circles indicate situations where Model 2A outperformed Model 2B, blue circles the opposite situation. Overlap score varies between 0 (no overlap) and 1 (complete overlap) and is proportional to the circles radius. L1-L6 = larval instars. P = pupa. NA = situation where the evaluation data consisted of a unique mean value, impeding the computation of an overlap score.

The proportion of individuals with slow and fast strategies within each population of *C. incommoda* was similar, and accounted for 37 to 59% of the population (18 to 29% for slow strategies and from 17 to 30% for fast strategies; Fig. 2). By accounting for these strategies, the predictive ability of Model 2B outperformed Model 2A in 89% of cases (Fig. 3, Supplementary Fig. S2), with an overlap varying between 0.09 and 0.89, whereas Model 2A outperformed Model 2B in 15% of the cases with an overlap ranging from 0.36 to 0.79. The overall model performance increase was consistent across life stages, with a slight decrease in prediction for the first two life stages. Both models performed similarly across rearing temperatures with the exception of the 30 °C population showing a lower mean overlap (0.5). Contrastingly, Model 1 outputs showed inaccurate predictions with a roots mean square error of phenological time reaching up to 25 days (Supplementary Table S3).

### Phenological variance and windows of interaction

**Figure 4:**
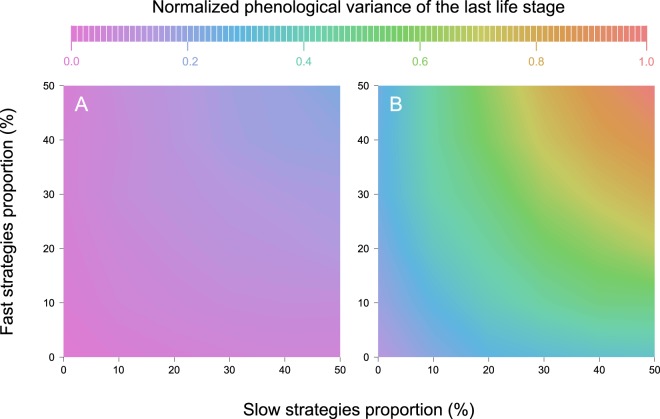
Predicted phenological variance (colours) for populations with different compositions of pace-of-life strategies (slow-fast developmental rates). Simulations were performed using Model 2B, with a virtual ectotherm species showing four (**A**), and eight (**B**) life stages. Development rates were generated under constant temperatures. Each value for the combination of slow and fast strategies represent the mean of 30 runs with 100 individuals.

We found that the phenological variance (i.e. the distribution of emergence dates) for the last life stage increased with the proportion of slow and fast strategies occurring within the populations, and with the number of total life stages (Fig. 4). A population from a species with four life stages composed of 10% of fast and 10% of slow individual strategies showed a normalized phenological variance of 0.1, whereas the normalized variance widened up to threefold when half of the individuals follows a fast strategy and the other half a slow strategy. A species with eight life stages experienced a similar threefold increase between 10–10% and 50–50% slow-fast strategies but with a wider overall variance (Fig. 4, Supplementary Fig. S3).

## Discussion

By independently following the individuals of *C. incommoda* during their whole life cycle, our study revealed the occurrence of pace-of-life (POL) strategies in the development of an ectothermic species. The slow and fast individuals accounted for a large part of the population (between 59 and 36%), with a roughly equal repartition between slow and fast (Fig. 2). As reported in other studies^32,33^, our population was not composed solely of strict slow and fast individuals but also included individuals showing an intermediate strategy with a phenotype close to the average of the population. This was due to individuals shifting between slow and fast strategies between life stages (Fig. 2). The lack of repeatability for performances (e.g. metabolism, behavior) across life stages has been observed in species of mammals^34^, birds^35^, reptiles^36^, amphibians^37^ and arthropods^38^. The individual POL strategies are more likely to switch between life stages with strong physiological differences, such as life stages before and after metamorphosis in amphibians or arthropods^37,38^. However, in the case of *C. incommoda*, we could not find any consistent patterns across individuals (Fig. 2). This result could be linked to the fact that the individual life-history has a strong impact on development compared to other performances that happen at a shorter timespan (e.g. locomotion). Moreover, because the life stage progress is linear, the inter-individual differences occurring at one life stage affect the outcome of the next life stage^10^. As the rearing temperatures were slightly fluctuating, differences in the temperatures experienced during early-life may have further affected the development during subsequent life stages. These peculiarities of development compared to other performances further support the development of thermal performance probability models in phenology studies.

A high predictive ability under several thermal contexts was shown in Models 2A and 2B (Fig. 3). Nevertheless, Model 2B performed better by integrating the slow-fast strategies stemming from the POL than Model 2A that accounted for intra-populational variability in development rates alone (Fig. 3). Few phenological models account for inter-individual differences in development to predict the range of phenological responses observed in nature^12,39,40^. To the best of our knowledge, this study is the first published to integrate the POL into an individual-based phenological modelling framework. The POL syndrome has been widely used to link an organism’s behavior with its metabolism^20,41,42^. Recently, is has been argued that this syndrome arises from lower level processes such as thermal physiology^25^ and that it is relevant at the individual level^14^. We showed that the inter-individual differences and the POL strategies are also relevant to predict phenology. The accurate quantitative predictions generated by our model strongly supports the importance of POL strategies as a driver of phenology^43^.

Our simulations suggest that the proportion of slow and fast individuals within a population of virtual ectotherms affects the phenology (c.f. distribution of individual emergence dates) of the population (Fig. 4). Larger proportions of slow or fast individuals increase the phenological variance, and the combination of slow and fast individuals further widen the variance (Fig. 4). The overall pattern arising from the simulation highlights a complementarity effect between the slow and fast strategies, which may have important ecological consequences. Indeed, the variance of a populational phenology delineates the potential interaction window with another species, which often change in strength and type across ontogeny^4^. Therefore, a phenological mismatch between ontogenies can result in weaker or shorter interactions^44^, or even change the type of interactions between species (e.g. predation to competition)^45^. In this context, we suggest that populations with a higher proportion of slow and fast individuals are expected to be more resilient to temporal mismatches between species, as their phenological window is wider. Our results also suggest that, for a given proportion of slow-fast individual, the number of life stages increases phenological variance (Fig. 4). As the number of life stages in ectothermic organisms shows a high variability across taxa^23^, temporal mismatch between species is likely to be stronger for species with fewer life stages. Overall, a population with a high proportion of slow or fast individuals are likely to offer a wider interaction window, especially for a species with a high number of life stages.

Even though the proposed model captures a large part of the observed variation across individuals and show a high predictive ability, it should be assessed under natural conditions as discrepancies may occur between phenology under controlled constant temperatures and natural fluctuating conditions^46–48^. Temperature fluctuations have different temporal scales, from diel changes up to climate change^49^. Spatial thermal heterogeneity due to biotic and abiotic factors also acts as a driver of thermal fluctuations experienced by small ectotherms moving across a mosaic of microclimates^50^. Therefore ectothermic animals can buffer thermal fluctuations by remaining in microhabitats near their optimal temperature^51,52^, or through behavioural thermoregulation^53^. A step further into the understanding and prediction of the inter-individual variations in phenology in natural habitat should integrate both these behavioural and spatial fluctuations in temperature.

## Methods

### Phenological models

Phenological predictions for a population of ectotherms (such as reptiles, amphibians or arthropods; Fig. 1) can be modelled by (i) determining the development response of the population to temperature, and (ii) accumulating the development rate over a temperature time-series^54^. A standard approach to studying the relationship between development and temperature is carried out by measuring the development rate across a range of environmental temperatures under controlled conditions (i.e. rearing experiments). The performance rate typically rises slowly with increasing temperatures until it reaches a maximum performance and then drops more or less abruptly (Fig. 1A). The accumulation process for each individual consists of (i) following a temperature time series with a specific time step (Fig. 1A1,B1), (ii) retrieving the development rate corresponding to the temperature at one discrete-time frame (triangles, squares and circles in Fig. 1A,A1), (iii) multiplying the development rate by the time step, (iv) accumulating the development rate until the next life stage is reached (e.g. juvenile lizard, tadpole and insect larva in Fig. 1A2), and (v) recording the time elapsed during the whole accumulation process (Fig. 1A2,B2,B3). The modelling steps are then repeated along the life stages (colours in Fig. 1) and individuals (to obtain the distributions of phenology in Fig. 1A2,B2,B3). Starting with this general framework, we propose increasingly complex models by deepening the underlying hypotheses behind the thermal characterization and the development accumulation processes. Our models account for phenological events during growth, but set aside the seasonal timing of such events.

### Model 1 – The commonly used thermal performance curve

Model 1 employs the commonly-used thermal performance curve (TPC) that allows estimating a continuous mean performance rate for the whole population^31^. The fitting process can be achieved by using simple or more complex models^31^. Once the TPC for development has been characterized, a development rate can be determined at any temperature within the thermal limits of the species (Fig. 1A1,A2). However, because the TPC offers a mean development rate response (Fig. 1A1), the dates of emergence for every life stages are identical across individuals (Fig. 1A2).

### Model 2A – Thermal performance probability

Model 2A is motivated by the fact that development occurs at longer time scales than other performances (e.g. locomotion). Indeed, for a given life stage, development rate cannot be measured at different temperatures on the same individual, which impedes the application of models quantifying within versus between individuals variances^55,56^. We chose a modelling approach easily integrated into the well-established performance accumulation models from TPC^27^. Instead of fitting a curve close to the mean of population measurements at various discrete temperatures, we propose to fit a mixture distribution of development responses at each discrete temperature^11^ (Supplementary Fig. S1). Finite mixture distributions are commonly used to identify sub-populations within a population^57^. These models assume that the sample observations randomly arise from two or more distributions with certain probabilities. Suppose that $X=({X}_{1},\,\mathrm{...},\,{X}_{n})$ is a random sample of size *n* from the mixed population, with a density function of *X*_*i*_ given by *f*(*X*_*i*_). In a generic case, *X* is assumed to have arisen from a mixture distribution with two components (*K* = 2) following normal distributions. Therefore, the density of *X*_*i*_ is given by:

1 $$f({X}_{i})=\sum _{k=1}^{K}{\lambda }_{k}N({\mu }_{k},\,{\sigma }_{k}^{2}),\,{\rm{with}}\,K=2$$

Where *λ*_*k*_ is the weight, *μ*_*k*_ is the mean, and ${\sigma }_{k}^{2}$ the variance of normal component *k*. We used an expectation–maximization (EM) algorithm to identify the mixture distribution parameters at the discrete known temperatures. The EM algorithm is decomposed in two steps, the expectation (E) step based on Baye’s theorem and the maximization (M) step (normalmixEM function from the mixtools R package)^57^. Several studies suggested that the distribution of development rates within a population follows a Weibull^40^ or a log-normal distribution^11^. However, following our pace-of-life hypothesis, we decided to fit a mixture of two normal distributions (i.e. slow and fast individuals). We found higher log likelihood scores for mixture distributions (i.e. bimodal distributions) than for Weibull and lognormal distributions in 42 out of 45 cases (Supplementary Table S2), therefore supporting our hypothesis.

To determine development rate distributions between the measured temperatures, we interpolated the normal distributions’ parameters (mean, standard deviation, and weight) to obtain a continuous response over the whole range of temperatures (Supplementary Fig. S1). Each parameter (*p*) from the mixture distributions was interpolated between two known development distributions at consecutive temperatures with the following expression:

2 $$p({T}_{x})={p}_{{T}_{1}}+({p}_{{T}_{2}}-{p}_{{T}_{1}})\cdot (1-\frac{{T}_{2}-{T}_{x}}{{T}_{2}-{T}_{1}}),\,{\rm{for}}\,{T}_{1} < {T}_{x} < {T}_{2}$$

Where *T*_1_ is the lower temperature at which parameter ${p}_{{T}_{1}}$ is known, *T*_2_ the higher temperature limit for the interpolation at which parameter ${p}_{{T}_{1}}$ is known, and *T*_*x*_ corresponds to the temperature at which the parameter (*p*(*T*_*x*_)) has to be determined. The parameters interpolation is then repeated for every parameter (*λ*_*k*_, *μ*_*k*_, and ${\sigma }_{k}^{2}$) and for the two normal components of the mixture distribution. We assumed that a linear interpolation would offer a sufficient fit if the number of rearing temperatures is high and evenly distributed across the thermal range. However, if the number of temperatures is low or does not incorporate the thermal limits, we advise using a non-linear interpolation based on one of the TPC models available, allowing to determine the thermal limits.

We named the resulting probability surface the Thermal Performance Probability (TPP), which is depicted graphically as a heat map with its area delimiting the response space, and the colour density representing the probability of the response (Fig. 1B, Supplementary Fig. S1). During the development accumulation process, more than one development response is possible at a given temperature, and the determination of this development rate is performed by drawing one value from the mixture distribution. This approach allows capturing a range of theoretical responses naturally occurring within a population (Fig. 1B). It results in a distribution of phenology for each life stage overlapping each other, with potential accelerations or buffering effects (Fig. 1B1,B2,B3).

### Model 2B – Thermal performance probability with pace-of-life strategies

A limitation of Model 2A is that one individual can exhibit development rates that vary from one extreme to another at two consecutive time frames within the same life stage, which may be unrealistic in natural populations, as suggested by the POL syndrome. To address this limitation, we implemented individual slow-fast strategies in Model 2B by dividing the population in three subpopulations with slow, fast and intermediate individuals. Development rate for the intermediate individual was drawn from the whole mixture distribution (identical as Model 2A), while the development rate for the slow and fast individuals was drawn from the first or second component distributions of the mixture distribution, respectively.

### Study case

To compare and illustrate the three models, we used development data from the quinoa moth (*Copitarsia incommoda*), and predicted its phenology distributions for each life stage. *C. incommoda* is an important pest of the quinoa crop (*Chenopodium quinoa*) and shows several characteristics representative of many species across the world. First, it thrives in a thermal environment with strong daily fluctuations (pers. obs.). Because a trade-off exists between adaptations at high and low temperatures, a high variability in performance responses to temperature is expected within and among the populations^27^. Second, the quinoa moth has numerous life stages (egg, six larval instars, pupa and adult) and each life stage has a particular thermal environment, which may trigger variable development rates across life stages^58^. Third, the quinoa moth lives in an environment with virtually unlimited food supply, which lowers the importance of this confounding factor on development. We reared each *C. incommoda* individual from each population in separate containers with identical thermal conditions to daily follow its development. By doing so, we were able to identify potential slow-fast strategies occurring among individuals and calibrated the models accordingly. We then compared models’ predictions with the observed phenology using a cross-validation approach.

To obtain data about the relationship between temperature and development, 960 eggs of *C. incommoda* were distributed among 8 rearing units with different temperatures spanning the range of temperatures in natural conditions (mean temperatures of 5.1, 12.6, 18.1, 20.2, 24.8, 30.0, 33.2, and 34.6 °C and standard deviations of 1.5, 1.3, 1.8, 2.2, 1.6, 1.0, 0.9, respectively, with 60 ± 5% relative humidity). We chose to create temperatures slightly fluctuating around a mean with low amplitude instead of strictly constant temperatures in order to stimulate the differences in development accumulation between individuals, without facing the issues of highly fluctuating temperatures^48^. Each rearing unit was checked once a day following a regular schedule, in which process life stage of each individual was individually followed (total of 4619 phenological measurements). Temperature in each rearing unit was measured every 30 minutes using temperature loggers (HOBO TidbiT v2 UTBI-001 from Onset) in order to feed the model with actual temperature time-series. Detailed information about the rearing experiments can be found in Rebaudo *et al*.^58^. The experiments complied with the Association for the Study of Animal Behaviour guidelines for the use of animals in research^59^.

To construct the models and evaluate their predictions, the development rate dataset for each temperature was split into two datasets following the cross-validation approach^7^: a calibration dataset (70% of the observations) and an evaluation dataset (30%). The three models were fitted on the development rates of the eight populations from the rearing units at eight mean temperatures. However, we used only the five temperatures where egg mortality was lower than 50% in the subsequent modelling steps (12.6, 18.1, 20.2, 24.8, 30.0 °C). We fitted Model 1 for each life stage using the Lactin-1 model^60^ to obtain the predicted phenologies for each life stage and to compare it to the observed one. All TPCs were computed using the devRate R package^61,62^. To construct Model 2B, we determined the empirical contribution of fast, slow, and intermediate individuals within each population by splitting the development range in two considering the median development value. We then followed each individual from egg until the sixth (last) larval instar, and recorded in which category (i.e. slow, or fast or intermediate relative to the median) it fell at each life stage. We decided to attribute a slow or fast strategy to an individual considering its strategy consistency across life stages. To do so, we determined the proportion of fast and slow individuals in each population by separating i) the fast individuals that stayed in the upper category for at least 5 out of 7 life stages (population reared at 13 °C = 30%, 18 °C = 17%, 21 °C = 19%, 26 °C = 20% and 30 °C = 22%) and ii) the slow individuals that stayed in the lower category for at least 5 out of 7 life stages (population reared at 13 °C = 29%, 18 °C = 19%, 21 °C = 18%, 26 °C = 24% and 30 °C = 22%). We focused on the larval stages because of the high mortality during the subsequent life stages. The three models were run along five independent temperature time-series (time-step of 24 h). The goodness-of-fit between predicted and observed phenology distributions were evaluated differently for Model 1 and Models 2 according to their outputs. Model 1 was evaluated by computing the Root Mean Squared Error between predicted and observed data as it does not account for variance in phenological outputs. Model 2A and 2B were evaluated by computing the overlap that accounts for the variance in the output. Measuring the overlap goes beyond comparing means and variances. An overlap of 1 means complete overlap between two distributions (i.e. perfect prediction), while an overlap value of 0 indicates an absence of overlap. The overlap estimator Dhat1 was used because most of our sample sizes were lower than 50^63^. The overlap were estimated for Model 2A and 2B using the overlapEst() function from the overlap R package^64^. All analyses were compiled with R 3.3.3.

### Exploring the thermal performance probability framework

To explore how Model 2B parameters affect phenology patterns, we generated development data for a virtual species with a varying number of life stages (4 and 8) and slow and fast proportion in the population (0, 10, 20, 30, 40, and 50% for each strategy). The generated population of 100 individuals had an inter-individual variability in development rate with an identical TPP across life stages. The model was performed on generated constant temperature time-series. We then assessed the combined consequences of these parameter values on the variance of the phenology distribution for the last life stage of the whole population, based on 30 runs for each combination of slow and fast strategies.

## Electronic supplementary material

Supplementary Information

## Data Availability

All data and computer source code used to calibrate and run the models are accessible with the 10.5281/zenodo.1421887.

## References

[1] CaraDonna PJ, Iler AM, Inouye DW (2014). Shifts in flowering phenology reshape a subalpine plant community. Proc. Natl. Acad. Sci..

[2] Parmesan C (2007). Influences of species, latitudes and methodologies on estimates of phenological response to global warming. Glob. Change Biol..

[3] Pilfold NW, McCall A, Derocher AE, Lunn NJ, Richardson E (2017). Migratory response of polar bears to sea ice loss: to swim or not to swim. Ecography.

[4] Forrest J, Miller-Rushing AJ (2010). Toward a synthetic understanding of the role of phenology in ecology and evolution. Philos. Trans. R. Soc. B Biol. Sci..

[5] Bellard C, Bertelsmeier C, Leadley P, Thuiller W, Courchamp F (2012). Impacts of climate change on the future of biodiversity: Biodiversity and climate change. Ecol. Lett..

[6] Wadgymar SM, Ogilvie JE, Inouye DW, Weis AE, Anderson JT (2018). Phenological responses to multiple environmental drivers under climate change: insights from a long‐term observational study and a manipulative field experiment. New Phytol..

[7] Chuine I, Régnière J (2017). Process-Based Models of Phenology for Plants and Animals. Annu. Rev. Ecol. Evol. Syst..

[8] Jackson MT (1966). Effects of Microclimate on Spring Flowering Phenology. Ecology.

[9] Dunne JA, Harte J, Taylor KJ (2003). Subalpine meadow flowering phenology responses to climate change: integrating experimental and gradient methods. Ecol. Monogr..

[10] Senner NR, Conklin JR, Piersma T (2015). An ontogenetic perspective on individual differences. Proc. R. Soc. B Biol. Sci..

[11] Régnière Jacques, Powell James A. (2013). Animal Life Cycle Models (Poikilotherms). Phenology: An Integrative Environmental Science.

[12] Régnière Jacques, Bentz Barbara J., Powell Jim A., St-Amant Rémi (2015). Individual-Based Modeling: Mountain Pine Beetle Seasonal Biology in Response to Climate. Simulation Modeling of Forest Landscape Disturbances.

[13] Ricklefs RE, Wikelski M (2002). The physiology/life-history nexus. Trends Ecol. Evol..

[14] Reale D (2010). Personality and the emergence of the pace-of-life syndrome concept at the population level. Philos. Trans. R. Soc. B Biol. Sci..

[15] Vitasse Y, Porté AJ, Kremer A, Michalet R, Delzon S (2009). Responses of canopy duration to temperature changes in four temperate tree species: relative contributions of spring and autumn leaf phenology. Oecologia.

[16] Gebhardt-Henrich S, Richner H (1998). Causes of growth variation and its consequences for fitness. Oxf. Ornithol. Ser..

[17] Benowitz-Fredericks ZM, Kitaysky AS (2005). Benefits and costs of rapid growth in common murre chicks Uria aalge. J. Avian Biol..

[18] Mueller P, Diamond J (2001). Metabolic rate and environmental productivity: well-provisioned animals evolved to run and idle fast. Proc. Natl. Acad. Sci..

[19] Lovegrove B (2003). The influence of climate on the basal metabolic rate of small mammals: a slow-fast metabolic continuum. J. Comp. Physiol. B.

[20] Careau V (2015). Energy expenditure and personality in wild chipmunks. Behav. Ecol. Sociobiol..

[21] Heflin LE (2013). Growth rates are related to production efficiencies in juveniles of the sea urchin Lytechinus variegatus. J. Mar. Biol. Assoc. U. K..

[22] Singh N, Mishra G, Omkar (2014). Does temperature modify slow and fast development in two aphidophagous ladybirds?. J. Therm. Biol..

[23] Moran NA (1994). Adaptation and constraint in the complex life cycles of animals. Annu. Rev. Ecol. Syst..

[24] Fusco G, Minelli A (2010). Phenotypic plasticity in development and evolution: facts and concepts. Philos. Trans. R. Soc. B Biol. Sci..

[25] Goulet CT, Thompson MB, Michelangeli M, Wong BBM, Chapple DG (2017). Thermal physiology: A new dimension of the pace-of-life syndrome. J. Anim. Ecol..

[26] Régnière, J. & Logan, J. A. Animal life cycle models. In *Phenology: an integrative environmental science* 237–254 (2003).

[27] Angilletta, M. J. *Thermal adaptation: a theoretical and empirical synthesis*. (Oxford University Press, 2009).

[28] Kingsolver JG (2011). Complex Life Cycles and the Responses of Insects to Climate Change. Integr. Comp. Biol..

[29] Feil R, Fraga MF (2012). Epigenetics and the environment: emerging patterns and implications. Nat. Rev. Genet..

[30] Burton T, Metcalfe NB (2014). Can environmental conditions experienced in early life influence future generations?. Proc. R. Soc. B Biol. Sci..

[31] Angilletta MJ (2006). Estimating and comparing thermal performance curves. J. Therm. Biol..

[32] Sæther B-E, Saether B-E (1987). The Influence of Body Weight on the Covariation between Reproductive Traits in European Birds. Oikos.

[33] McCauley SJ (2008). Slow, fast and in between: habitat distribution and behaviour of larvae in nine species of libellulid dragonfly. Freshw. Biol..

[34] Russell GA, Chappell MA (2006). Is BMR repeatable in deer mice? Organ mass correlates and the effects of cold acclimation and natal altitude. J. Comp. Physiol. B.

[35] Chappell MA, Bachman GC, Odell JP (1995). Repeatability of Maximal Aerobic Performance in Belding’s Ground Squirrels, Spermophilus beldingi. Funct. Ecol..

[36] Clusella Trullas S, Terblanche JS, van Wyk JH, Spotila JR (2007). Low repeatability of preferred body temperature in four species of Cordylid lizards: Temporal variation and implications for adaptive significance. Evol. Ecol..

[37] Watkins TB (1997). The effect of metamorphosis on the repeatability of maximal locomotor performance in the Pacific tree frog Hyla regilla. J. Exp. Biol..

[38] Wexler Y, Subach A, Pruitt JN, Scharf I (2016). Behavioral repeatability of flour beetles before and after metamorphosis and throughout aging. Behav. Ecol. Sociobiol..

[39] de Jong, G. & van der Have, T. Temperature Dependence of Development Rate, Growth Rate and Size: From Biophysics to Adaptation. In *Phenotypic plasticity of insects: mechanisms and consequences*. (Whitman, D., 2009).

[40] Wagner TL, Wu H-I, Sharpe PJ, Coulson RN (1984). Modeling distributions of insect development time: a literature review and application of the Weibull function. Ann. Entomol. Soc. Am..

[41] Hall ML (2015). Animal personality and pace-of-life syndromes: do fast-exploring fairy-wrens die young?. Front. Ecol. Evol..

[42] Biro PA, Stamps JA (2010). Do consistent individual differences in metabolic rate promote consistent individual differences in behavior?. Trends Ecol. Evol..

[43] Houlahan JE, McKinney ST, Anderson TM, McGill BJ (2017). The priority of prediction in ecological understanding. Oikos.

[44] Høye TT, Post E, Schmidt NM, Trøjelsgaard K, Forchhammer MC (2013). Shorter flowering seasons and declining abundance of flower visitors in a warmer Arctic. Nat. Clim. Change.

[45] Yang LH, Rudolf VHW (2010). Phenology, ontogeny and the effects of climate change on the timing of species interactions. Ecol. Lett..

[46] Stillman JH (2003). Acclimation capacity underlies susceptibility to climate change. Science.

[47] Nagano AJ (2012). Deciphering and Prediction of Transcriptome Dynamics under Fluctuating Field Conditions. Cell.

[48] Colinet H, Sinclair BJ, Vernon P, Renault D (2015). Insects in Fluctuating Thermal Environments. Annu. Rev. Entomol..

[49] Dillon ME (2016). Life in the Frequency Domain: the Biological Impacts of Changes in Climate Variability at Multiple Time Scales. Integr. Comp. Biol..

[50] Woods HA, Dillon ME, Pincebourde S (2015). The roles of microclimatic diversity and of behavior in mediating the responses of ectotherms to climate change. J. Therm. Biol..

[51] Maclean IMD, Suggitt AJ, Wilson RJ, Duffy JP, Bennie JJ (2017). Fine-scale climate change: modelling spatial variation in biologically meaningful rates of warming. Glob. Change Biol..

[52] Faye E, Rebaudo F, Carpio C, Herrera M, Dangles O (2017). Does heterogeneity in crop canopy microclimates matter for pests? Evidence from aerial high-resolution thermography. Agric. Ecosyst. Environ..

[53] Kearney M, Shine R, Porter WP (2009). The potential for behavioral thermoregulation to buffer “cold-blooded” animals against climate warming. Proc. Natl. Acad. Sci..

[54] Pincebourde S, Sinoquet H, Combes D, Casas J (2007). Regional climate modulates the canopy mosaic of favourable and risky microclimates for insects. J. Anim. Ecol..

[55] van de Pol M, Wright J (2009). A simple method for distinguishing within- versus between-subject effects using mixed models. Anim. Behav..

[56] Violle C (2012). The return of the variance: intraspecific variability in community ecology. Trends Ecol. Evol..

[57] Benaglia T, Chauveau D, Hunter D, Young D (2009). mixtools: An R package for analyzing finite mixture models. J. Stat. Softw..

[58] Rebaudo F, Struelens Q, Callizaya Condori F, Quispe R (2017). Relationship between temperature and development rate of Copitarsia incommoda (Lepidoptera: Noctuidae) in the Bolivian Andes. Appl. Entomol. Zool..

[59] Buchanan K (2012). Guidelines for the treatment of animals in behavioural research and teaching. Anim. Behav..

[60] Lactin DJ, Holliday NJ, Johnson DL, Craigen R (1995). Improved rate model of temperature-dependent development by arthropods. Environ. Entomol..

[61] R Development Core Team. *R: A language and environment for statistical computing*. (R Foundation for Statistical Computing, 2018).

[62] Rebaudo F, Struelens Q, Dangles O (2018). Modelling temperature‐dependent development rate and phenology in arthropods: The devRate package for R. Methods Ecol. Evol..

[63] Schmid F, Schmidt A (2006). Nonparametric estimation of the coefficient of overlapping—theory and empirical application. Comput. Stat. Data Anal..

[64] Meredith, M. & Ridout, M. Overview of the overlap package. *R-CRAN* Available at, http://cran.at.r-project.org/web/packages/overlap/vignettes/overlap.pdf (2017).

