# Supplementary material for: Thermal pace-of-life strategies improve phenological predictions in ectotherms
Source: Sci Rep. 2018 Oct 26;8:15891. doi: 10.1038/s41598-018-34274-1 (PMC6203799; doi:10.1038/s41598-018-34274-1)
Supplement: Supplementary file 1 — Supplementary Information [file 41598_2018_34274_MOESM1_ESM.pdf]

Thermal pace-of-life strategies improve phenological predictions in ectotherms

Quentin Struelens[1,2,*], François Rebaudo[3], Reinaldo Quispe[4], Olivier Dangles[2,5]

[1]Muséum National d'Histoire Naturelle, Sorbonne Universités, Paris, France.

[2]Institut de Recherche pour le Développement, Centre d'Ecologie Fonctionnelle et Evolutive, UMR 5175, CNRS, Université de Montpellier, Université Paul Valéry, Montpellier, EPHE, IRD, Montpellier, France.

[3]Institut de Recherche pour le Développement, UMR EGCE-Université Paris Sud-CNRS-IRD-Paris Saclay, Gif-sur-Yvette, France.

[4]PROINPA, La Paz, Bolivia.

[5]Department of Ecology and Evolutionary Biology, Cornell University, Ithaca, NY.

[*]Corresponding author:

Quentin Struelens

Muséum National d'Histoire Naturelle

Sorbonne Universités

57 rue Cuvier

75005 Paris, France

E-mail: quentin.struelens@ird.fr

Supplementary Information

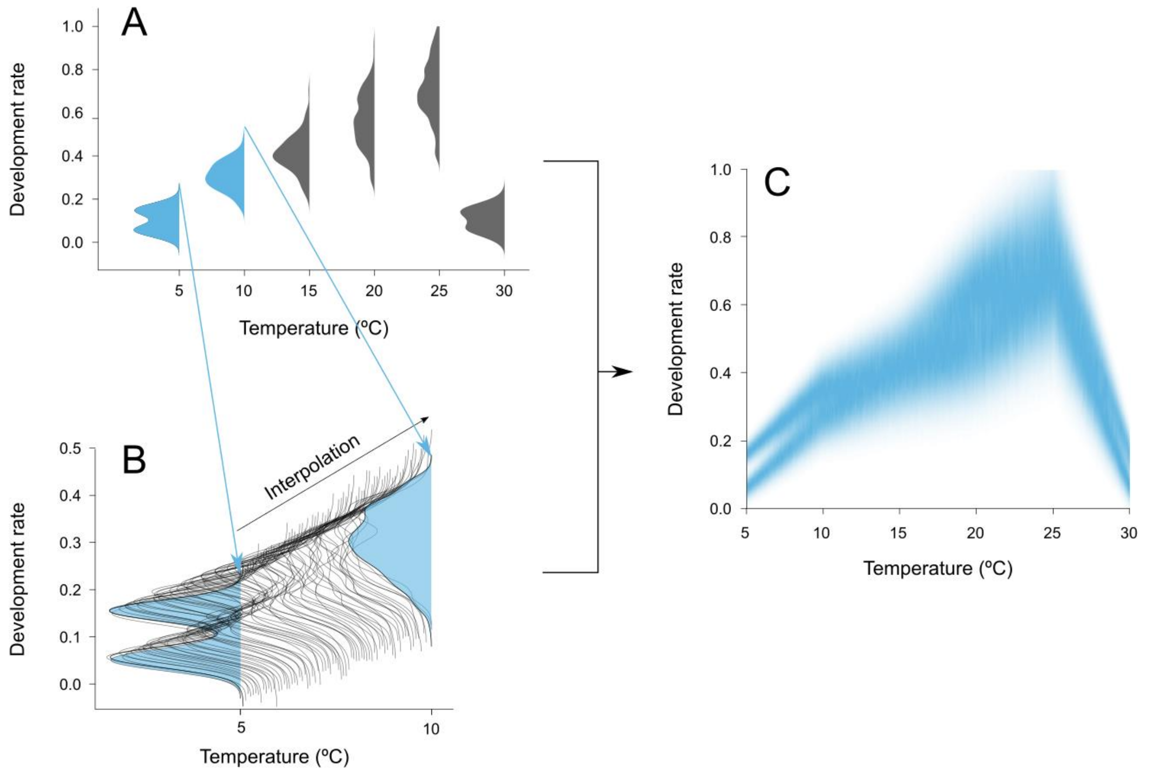

Supplementary Figure S1. Schema depicting the construction of the continuous thermal performance probability from performances rates at discrete temperatures based on generated data. A) A mixture distribution of two normal components is fitted on the empirical development rate at each discrete rearing temperature. B) Linear interpolation of the mixture distributions parameters (mean, standard deviation and weight) between two consecutive rearing temperatures. This process is repeated over all the temperatures to obtain C).

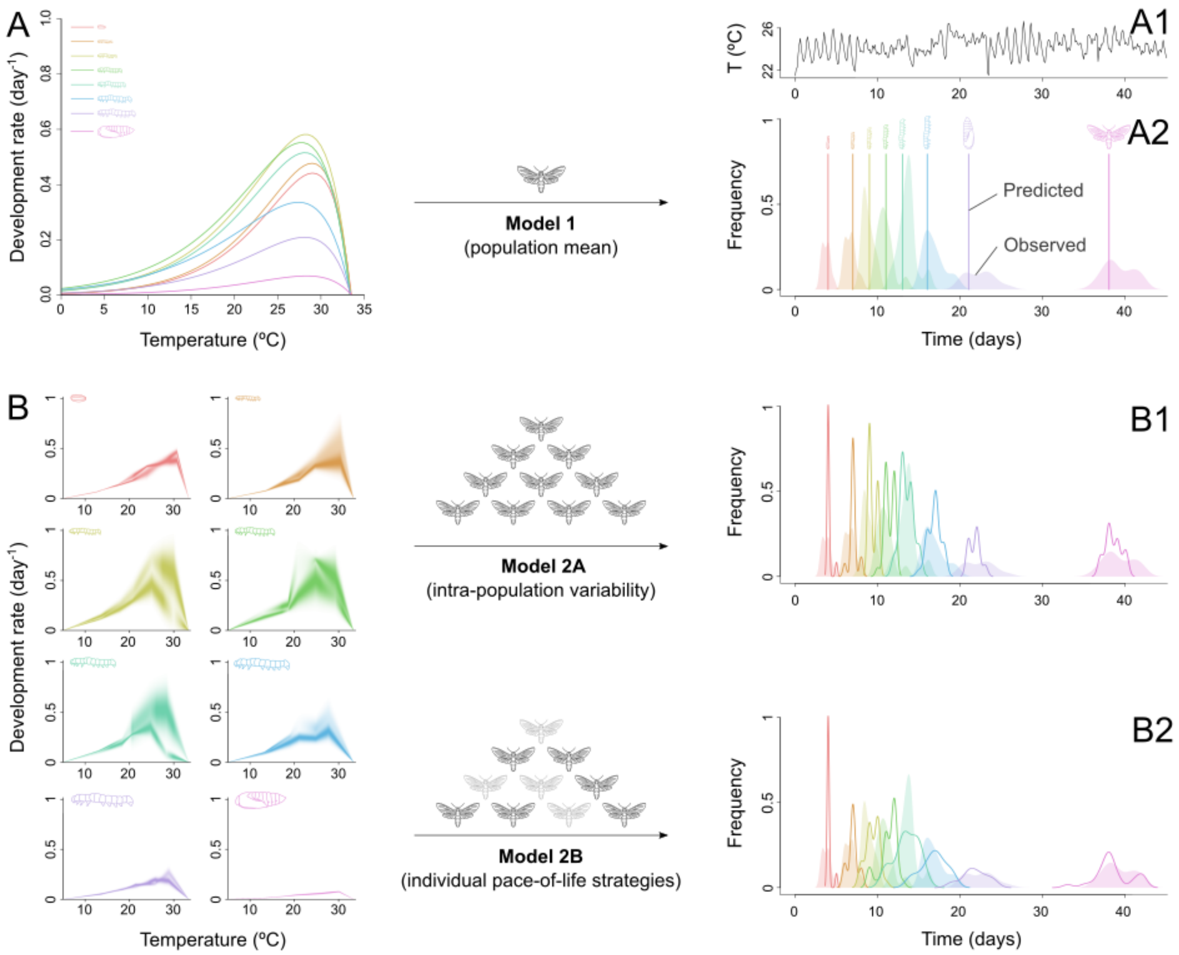

Supplementary Figure S2. Model construction and predictions of the phenology of *Copitarsia incommoda* based on development data from rearing at 25ºC mean temperature. Model 1 fits a mean thermal performance curve (TPC) between temperature and species' development rates for the eight life stages (egg, 1-6 larval instars and pupa; A), and then applies the TPC over a temperature time series (A1) to obtain the phenology of the eight life stages (1-6 larval instars, pupa and adult; A2). Model 2A constructs a thermal performance probability (TPP) surface for the eight life stages (egg, 1-6 larval instars and pupa; B) and then applies the TPP over the same temperature time-series (A1) to obtain the population distribution of the phenology over time, resulting in overlapping life stages (1-6 larval instars, pupa and adult; B1). Model 2B applies the same TPP than Model 2A (B) over the temperature time series (A1) but takes into account slow-fast strategies in development among individuals (see text for details) to obtain population distribution of the phenology over time (B2). Plain polygons represent observed phenology distributions (from evaluation dataset) while solid lines represent phenology distributions predicted by the models (from calibration dataset).

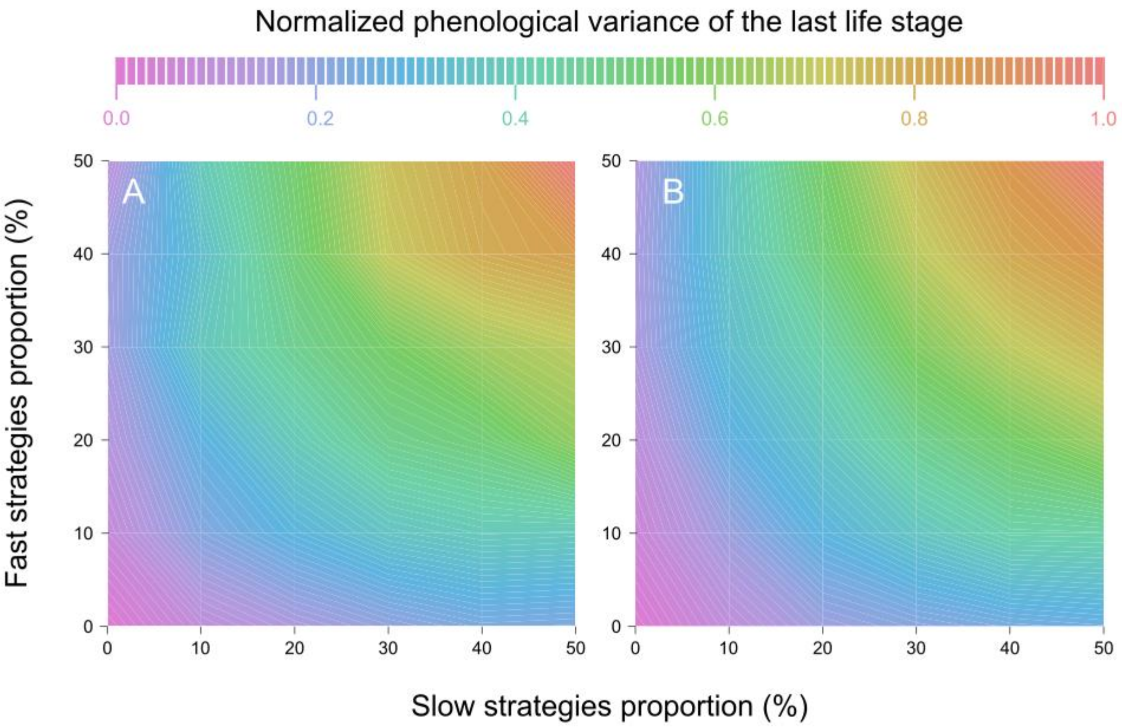

Supplementary Figure S3. Predicted phenological variance (colors) for populations with different compositions of pace-of-life strategies (slow-fast developmental rates). Simulations were performed using Model 2B, with a virtual ectotherm species showing four (A), and eight (B) life stages. Phenological variance has been normalized for each population independently to highlight the similarity in complementary pattern between life stages. Development rates were generated under constant temperatures. Each value for the combination of slow and fast strategies represent the mean of 30 runs with 100 individuals.

Supplementary Table S1. Examples of phenological variance between individuals of the same species naturally occurring under various conditions (non-exhaustive list). The longest timing difference refers to the period in days between the first and last individuals entering the phenological event. Values were not directly available from the studies, and were therefore estimated from figures within these studies.

**Phyla**	**Phenological event**	**Family**	**Species**	**Context**	**Timing difference (days)**	**Reference**
**Amphibians**	Metamorphosis	Ranidae	*Rana boylii*	Natural conditions	8	65
**Arthropods**	Life stage emergence	Curculionidae	*Dendroctonus ponderosae*	Controlled conditions	2.15	66
		Coccinellidae	*Menochilus sexmaculatus*	Controlled conditions	7	22
		Geometridae	*Pseudocoremia suavis*	Controlled conditions	28	67
		Sesarmidae	*Sesarma cinereum*	Controlled conditions	14	68
	Diapause completion	Tephritidae	*Rhagoletis mendax*	Controlled conditions	58	69
**Fishes**	Life stage emergence	Pleuronectidae	*Hippoglossus hippoglossus*	Controlled conditions	3	70
		Clupeidae	*Clupea harengus*	Controlled conditions	46	71
		Caligidae	*Caligus rogercresseyi*	Controlled conditions	10	72
		Scombridae	*Thunnus thynnus*	Controlled conditions	8	73
		Pleuronectidae	*Pleuronectes platessa*	Controlled conditions	35	74
	Migration	Salmonidae	*Oncorhynchus kisutch*	Controlled conditions	184	75
**Plants**	Development	Poaceae	*Echinochloa crus-galli*	Experimental plot	80	76
	Flowering	Sapindaceae	*Cupania guatemalensis*	Experimental plot	18	77
		Rhamnaceae	*Discaria toumatou*	Natural conditions	10	78
	Germination	Plantaginaceae	*Veronica arvensis*	Controlled conditions	25	79
		Fabaceae	*Trifolium subterraneum*	Experimental plot	14	80

Supplementary Table S2. Comparison of log-likelihood scores of the fits of Weibull, log-normal and mixture (bimodal) distributions for the populational development rate of *C. incommoda*. The 45 populations arise from nine life stages reared at five different temperatures. Bold values highlight the highest log likelihood score for each population.

		13ºC	18ºC	20ºC	25ºC	30ºC
Eggs	Weibull	489,54	432,18	272,20	190,41	162,87
	Lognormal	511,63	435,25	278,08	190,28	166,99
	Bimodal	**528,31**	**442,23**	**281,38**	**200,79**	**177,42**
Larva 1	Weibull	229,25	208,49	76,02	25,02	**3,26**
	Lognormal	200,60	212,40	**94,07**	29,19	-12,21
	Bimodal	**245,69**	**214,52**	90,91	**29,87**	2,86
Larva 2	Weibull	158,11	193,48	75,13	47,47	17,19
	Lognormal	146,63	195,48	104,93	48,89	7,55
	Bimodal	**161,42**	**200,81**	**121,22**	**57,59**	**21,14**
Larva 3	Weibull	128,36	213,64	58,97	45,60	27,07
	Lognormal	137,63	212,61	82,78	46,81	24,15
	Bimodal	**138,43**	**221,24**	**94,35**	**49,33**	**28,51**
Larva 4	Weibull	179,01	235,70	110,12	34,21	16,81
	Lognormal	177,72	250,95	119,54	43,10	13,15
	Bimodal	**180,01**	**254,38**	**123,57**	**53,19**	**24,37**
Larva 5	Weibull	128,41	178,07	114,19	121,97	26,49
	Lognormal	149,25	183,66	144,13	124,44	38,55
	Bimodal	**166,62**	**186,33**	**166,21**	**129,03**	**46,55**
Larva 6	Weibull	**229,67**	340,33	292,18	210,61	101,61
	Lognormal	224,90	365,40	288,53	205,13	101,19
	Bimodal	229,15	**379,04**	**294,33**	**220,84**	**103,94**
Pupa	Weibull	223,09	495,04	297,28	309,23	76,45
	Lognormal	231,15	498,60	297,82	311,72	79,63
	Bimodal	**234,61**	**502,32**	**304,41**	**314,83**	**79,69**
Adult	Weibull	86,90	134,24	128,46	162,65	15,50
	Lognormal	88,67	136,29	136,29	164,97	16,07
	Bimodal	**91,10**	**140,02**	**140,97**	**167,23**	**22,16**

Supplementary Table S3. Root mean square errors between observed and predicted phenologies from Model 1 in days. The comparison originates from a cross-validation process, with an evaluation dataset (30% of all data) and a calibration dataset used to predict the phenologies (70%).

Life stage	13ºC	18ºC	20ºC	25ºC	30ºC
L1	0,93	0,50	0,66	0,54	1,49
L2	2,20	1,40	1,11	0,72	3,12
L3	2,55	1,17	1,20	0,69	5,10
L4	1,98	1,23	1,51	0,95	5,76
L5	2,41	2,14	1,71	1,21	5,46
L6	4,48	2,14	1,73	1,50	4,64
Pupa	6,70	1,57	1,95	2,18	5,67
Adults	25,41	2,46	5,88	2,12	6,65

References Supplementary Material

22. Singh, N., Mishra, G. & Omkar. Does temperature modify slow and fast development in two aphidophagous ladybirds? *J. Therm. Biol.* **39,** 24–31 (2014).
65. Wheeler, C. A., Bettaso, J. B., Ashton, D. T. & Welsh, H. H. Effects of Water Temperature on Breeding Phenology, Growth, and Metamorphosis of Foothill Yellow-Legged Frogs (*Rana boylii*): A Case Study of the Regulated Mainstem and Unregulated Tributaries of California's Trinity River. *River Res. Appl.* **31,** 1276–1286 (2015).
66. Bentz, B. J., Logan, J. A. & Amman, G. D. Temperature-dependent development of the mountain pine beetle (Coleoptera: Scolytidae) and simulation of its phenology. *Can. Entomol.* **123,** 1083–1094 (1991).
67. Berndt, L., Brockerhoff, E. G., Jactel, H., Weiss, T. & Beaton, J. Biology and rearing of Pseudocoremia suavis, an endemic looper (Lepidoptera: Geometridae) with a history of outbreaks on exotic conifers. *N. Z. Entomol.* **27,** 73–82 (2004).
68. Costlow, J. D., Bookhout, C. G. & Monroe, R. The effect of salinity and temperature on larval development of Sesarma cinereum (Bosc) reared in the laboratory. *Biol. Bull.* **118,** 183–202 (1960).
69. Teixeira, L. A. F. & Polavarapu, S. Heat Stress Inhibits the Completion of Pupal Diapause in Rhagoletis mendax (Diptera: Tephritidae). *Ann. Entomol. Soc. Am.* **98,** 197–204 (2005).

70. Blaxter, J., Danielssen, D., Moksness, E. & Øiestad, V. Description of the early development of the halibut Hippoglossus hippoglossus and attempts to rear the larvae past first feeding. *Mar. Biol.* **73,** 99–107 (1983).
71. Gamble, J. C., MacLachlan, P. & Seaton, D. D. Comparative growth and development of autumn and spring spawned Atlantic herring larvae reared in large enclosed ecosystems. *Mar. Ecol. Prog. Ser.* **26,** 19–33 (1985).
72. González, L. & Carvajal, J. Life cycle of Caligus rogercresseyi, (Copepoda: Caligidae) parasite of Chilean reared salmonids. *Aquaculture* **220,** 101–117 (2003).
73. Kaji, T., Tanaka, M., Takahashi, Y., Oka, M. & Ishibashi, N. Preliminary observations on development of Pacific bluefin tuna Thunnus thynnus (Scombridae) larvae reared in the laboratory, with special reference to the digestive system. *Mar. Freshw. Res.* **47,** 261–269 (1996).
74. Ryland, J. S. Observations on the development of larvae of the plaice, Pleuronectes platessa L., in aquaria. *ICES J. Mar. Sci.* **30,** 177–195 (1966).
75. Sundström, L. F., Löhmus, M. & Devlin, R. H. Migration and growth potential of coho salmon smolts: implications for ecological impacts from growth-enhanced fish. *Ecol. Appl.* **20,** 1372–1383 (2010).
76. Norris, R. F. Morphological and phenological variation in barnyardgrass (*Echinochloa crus-galli*) in California. *Weed Sci.* **44,** 804–814 (1996).
77. Bawa, K. S. The Reproductive Biology of *Cupania guatemalensis* Radlk. (Sapindaceae). *Evolution* **31,** 52–63 (1977).
78. Primack, R. B. Variation in the Phenology of Natural Populations of Montane Shrubs in New Zealand. *J. Ecol.* **68,** 849–862 (1980).
79. Baskin, J. M. & Baskin, C. C. Germination Ecology of *Veronica Arvensis*. *J. Ecol.* **71,** 57–68 (1983).
80. Black, J. N. & Wilkinson, G. N. The role of time of emergence in determining the growth of individual plants in swards of subterranean clover (Trifolium subterraneum L.). *Aust. J. Agric. Res.* **14,** 628–638 (1963).
